# Bioactive Hydroxyapatite-Magnesium Phosphate Coatings Deposited by MAPLE for Preventing Infection and Promoting Orthopedic Implants Osteointegration

**DOI:** 10.3390/ma15207337

**Published:** 2022-10-20

**Authors:** Denisa Alexandra Florea, Valentina Grumezescu, Alexandra Cătălina Bîrcă, Bogdan Ștefan Vasile, Andrei Iosif, Cristina Chircov, Miruna S. Stan, Alexandru Mihai Grumezescu, Ecaterina Andronescu, Mariana Carmen Chifiriuc

**Affiliations:** 1 Department of Science and Engineering of Oxide Materials and Nanomaterials, Politehnica University of Bucharest, 011061 Bucharest, Romania; 2 National Institute for Lasers, Plasma and Radiation Physics, 077125 Magurele, Romania; 3 Research Institute of the University of Bucharest—ICUB, University of Bucharest, 050657 Bucharest, Romania; 4 Department of Biochemistry and Molecular Biology, Faculty of Biology, University of Bucharest, 050095 Bucharest, Romania; 5 Academy of Romanian Scientists, Ilfov No. 3, 050044 Bucharest, Romania; 6 Department of Microbiology, Faculty of Biology, University of Bucharest, 060101 Bucharest, Romania; 7 The Romanian Academy, Calea Victoriei 25, District 1, 010071 Bucharest, Romania

**Keywords:** *Staphylococcus aureus* biofilm, antibiotic-containing coatings, ceftriaxone, BMP4, osteoblasts, MAPLE

## Abstract

In this study, we used the matrix-assisted pulsed laser evaporation (MAPLE) technique to obtain hydroxyapatite (Ca_10_(PO_4_)_6_(OH)_2_) and magnesium phosphate (Mg_3_(PO_4_)_2_) thin coatings containing bone morphogenetic protein (BMP4) for promoting implants osteointegration and further nebulized with the antibiotic ceftriaxone (CXF) to prevent peri-implant infections. The samples were characterized by X-ray diffraction (XRD), scanning electron microscopy (SEM), transmission electron microscopy (TEM), selected area electron diffraction (SAED), infrared microscopy (IRM) and Fourier-transform infrared spectroscopy (FT-IR). Furthermore, the antimicrobial properties were evaluated on *Staphylococcus aureus* biofilms and the cytocompatibility on the MC3T3-E1 cell line. The obtained results proved the potential of the obtained coatings for bone implant applications, providing a significant antimicrobial and antibiofilm effect, especially in the first 48 h, and cytocompatibility in relation to murine osteoblast cells.

## 1. Introduction

Considering the hundreds of millions of individuals affected by bone disorders [1,2,3] and the subsequent significant increase in the necessity of bone implants [3,4,5], one of the current research focuses is to obtain improved implants with increased osseointegration, osteoinduction, and vascularization properties, as well as mechanical stability, good corrosion resistance, and cost-effectiveness [6,7,8,9]. The commercially available implants are generally fabricated from metal, ceramic, and/or polymer composites (i.e., Ti, Co-Cr, Fe, and their alloys, Al_2_O_3_, ZrO_2,_ hydroxyapatite (HA), β-tricalcium phosphate, bioglasses) [10,11,12,13,14,15,16,17].

The main challenge for future research is to achieve a histologically and biomechanically stable implant-tissue interface [18]. To achieve this goal, one of the most promising research directions in implantology is represented by the development of bioactive coatings with suitable biological and mechanical properties.

HA is a major bone constituent, therefore, its natural and synthetic forms are commonly used as a coating on metallic implants and to replace hard tissues [19,20]. Though calcium-based apatites have been investigated over time and showed promising results as bone substitutes, their poor mechanical properties impair their use for load-bearing implants [21]. One of the solutions to improve the HA mechanical properties is the substitution of calcium with magnesium ions [22,23,24,25,26,27]. Thus, magnesium phosphates have become an attractive lead for orthopedics, exhibiting, besides their significantly higher mechanical resistance, other advantages such as improved bone fragment fixation, higher dissolution rates, and bone ingrowth acceleration compared to calcium phosphates [28,29,30,31,32]. Moreover, HA and Mg_3_(PO_4_)_2_ could contribute to achieving roughened surfaces that promote osseointegration [33].

The matrix-assisted pulsed laser evaporation (MAPLE) method has multiple advantages, such as it allows the preparation of coatings containing organic materials [34,35,36,37,38] such as growth factors and antibiotics, with controlled thickness, homogeneity, etc.

Thus, the purpose of our study was to obtain bioactive coatings prepared by MAPLE capable of releasing a growth factor to increase implant osseointegration and containing a large-spectrum antibiotic to provide increased resistance to microbial colonization.

In our study, we opted to incorporate in the coatings the bone morphogenetic protein 4 (BMP4) based on its previously established capacity to promote the proliferation and differentiation of mesenchymal and bone marrow basal cells with the formation of bone tissue, as well as of fibroblasts into osteoblasts and of myoblasts into chondrocytes, thus inducing matrix calcification [39]. Moreover, previous studies have shown that HA micro/nanoporous structures could successfully load and release BMP4 [40,41,42,43].

To provide antimicrobial protection, we chose to load the third-generation cephalosporin antibiotic ceftriaxone (CXF) in the obtained coatings. It is well known that one of the major causes of implant failure is represented by the periprosthetic orthopedic infections, either exogenous; the pathogenic bacteria from the personnel’s skin or environmental surfaces being introduced via the surgical incision; or endogenous, the bacterial cell from the patient’s body accessing the implant site by hematogenous dissemination [44]. The microbial cells colonizing the implant surface could develop mature biofilms that are resistant to available therapeutic options and could lead to chronic and hard-to-diagnose infections [16,17]. Previous studies have shown that biodegradable mineral-based bone cements could incorporate and deliver high local concentrations of antibiotics, which otherwise cannot be achieved systemically due to their toxicity. Although the antibiotic is generally released by diffusion and thus, the release rate is hard to control, even a short window of high local antibiotic concentration might be sufficient to prevent biofilm initiation by killing the planktonic cells from the vicinity of the implant surface or even to treat experimental osteomyelitis caused methicillin-resistant *Staphylococcus aureus* [45,46,47,48].

## 2. Materials and Methods

### 2.1. Materials

Calcium chloride (CaCl_2_), sodium phosphate dibasic (Na_2_HPO_4_), magnesium chloride hexahydrate (MgCl_2_·6H_2_O), phosphoric acid (H_3_PO_4_), sodium hydroxide (NaOH), chloroform (CHCl_3_), and dimethyl sulfoxide (99%, DMSO), bone morphogenetic protein (BMP4) and ceftriaxone (CXF) were purchased from Sigma-Aldrich Merck (Darmstadt, Germany). All chemicals were of analytical purity and used with no further purification.

### 2.2. Synthesis of Nanoparticles

The HA nanoparticles were synthesized by the co-precipitation process of the calcium and phosphate precursors. Briefly, CaCl_2_ and Na_2_HPO_4_ were separately solubilized into 200 mL of deionized water at room temperature. Subsequently, the Na_2_HPO_4_ solution was added dropwise into the CaCl_2_ solution under continuous magnetic stirring, followed by the addition of NaOH to adjust the pH to 9.5. The obtained precipitate was filtered, washed with deionized water, and dried at room temperature for 24 h. The resulting powder was calcined at 650 °C for 2 h.

The synthesis of magnesium phosphate—Mg_3_(PO_4_)_2_, (MgP) involved the solubilization of MgCl_2_·6H_2_O and H_3_PO_4_ in 200 mL H_2_O, followed by the dropwise addition of the H_3_PO_4_ solution into MgCl_2_·6H_2_O solution. The pH was adjusted from 2 to 9 by adding NaOH under a cooling bath reaction for 24 h. The so-obtained gel was filtered, washed, dried at room temperature, and calcined at 850 °C for 6 h.

### 2.3. Coatings Synthesis by MAPLE Technique

MAPLE targets were prepared by freezing at liquid nitrogen temperature a solution of 4% (*w*/*v*) HA, Mg_3_(PO_4_)_2_, (1:1 wt%) and 50 ng/mL BMP4 in DMSO. A KrF* (λ = 248 nm, τFWHM = 25 ns) (COMPexPro 205 Lambda Physics-Coherent) excimer laser beam impinged the target at 300, 400, and 500 mJ/cm^2^ laser fluences, with a repetition rate of 15 Hz and for 50,000 pulses. During the laser irradiation, the cryogenic target was maintained at a temperature of ∼173 K by constant cooling with liquid nitrogen, rotated at a rate of 0.4 Hz, and upheld at a 4 cm distance from substrates. All depositions on the titanium disks were conducted at room temperature and with a background pressure of 1 Pa.

After HA/MgP/BMP4 coatings were prepared by MAPLE, the surface was further nebulized with a solution consisting of a mix between 100 µg CXF and 1 mL CHCl_3_. Each sample was nebulized with the same amount of CXF.

### 2.4. Physico-Chemical Characterization Methods

#### 2.4.1. X-ray Diffraction (XRD)

The XRD analysis was performed on a PANalytical Empyrean diffractometer (PANalytical, Almelo, The Netherlands) equipped with CuK_α_ radiation (λ = 1.541874 Å), a hybrid 2 × Ge (220) monochromator for Cu and a parallel plate collimator on the PIXcel3D detector. Measurements were taken at room temperature between 2θ angles of 10° and 80°, with a step size of 0.04° and time per step of 3 s. Data processing was performed using the HighScore Plus software (version 3.0, PANalytical, Almelo, The Netherlands).

#### 2.4.2. Transmission Electron Microscopy (TEM). Selected Area Electron Diffraction (SAED)

Small amounts of HA and MgP powders were dispersed into deionized water, and 10 µL was placed on a 400-mesh lacey carbon-coated copper grid at room temperature. The TEM micrographs and SAED patterns were acquired using a TecnaiTM G2 F30 S-TWIN transmission electron microscope (purchased from FEI, Hillsboro, OR, USA) operated at 300 kV.

#### 2.4.3. Infrared Microscopy (IRM). Fourier-Transform Infrared Spectroscopy (FT-IR)

IR spectra and IR maps were obtained using FT-IR Nicolet 6700 equipment (Thermo Nicolet, Madison, WI, USA). For each sample, 32 scans were executed between 4000–1000 cm^−1^, at a 4 cm^−1^ resolution. The Omnic 8.2. software was used to process the recorded data.

#### 2.4.4. Scanning Electron Microscopy (SEM)

The morphology and dimension of the nanostructures were investigated using an Inspect F50 high-resolution scanning electron microscope (Thermo Fisher—former FEI, Eindhoven, The Netherlands). The powders were placed on a carbon tape, and the images were acquired by recording the resulting secondary electrons at an energy of 30 keV. For cross-section analysis, the sample was prepared using a diamond knife.

### 2.5. Cytocompatibility Analysis

Murine osteoblasts (MC3T3-E1 cell line, SigmAldrich, Saint Louis, MO, USA) were used to evaluate the cellular behavior in contact with HA/MgP/BMP4 nanostructures by fluorescence microscopy, using specific staining for actin cytoskeleton and nucleus, as well as by quantitative methods to assess cell viability and membrane integrity. For this purpose, the coatings were deposited on transparent glass in order to allow the microscopic examination of the mammalian cells. Prior to use, all samples (glass slides with nanocoating and those without nanocoating used as control) were subjected to UV sterilization for 1 h. Furthermore, the cells were seeded on the surface of slides at a density of 5 × 10^4^ cells/cm^2^ and kept for 24 h and 72 h in the incubator at 37 °C in a humid atmosphere with 5% CO_2_. Phalloidin-fluorescein isothiocyanate (FITC) staining protocol was followed to evaluate the actin filaments under fluorescence microscopy and determine the changes at the cytoskeleton level after 24 h. After incubation, cells were fixed with 4% paraformaldehyde and permeabilized with 0.5% Triton X-100 and 1% bovine serum albumin (SigmAldrich, Saint Louis, MO, USA). Staining was performed with phalloidin-FITC and 4′,6-diamidino-2-phenylindole (DAPI) for actin and nuclei, respectively. An Olympus IX71 inverted microscope (Olympus, Tokyo, Japan) was used to visualize the images.

Cellular viability was determined using the (3-(4,5-dimethylthiazol-2-yl)-2,5-diphenyltetrazolium bromide (MTT) solution. After removing the media from 24-well plates, the osteoblasts were incubated for 3 h with 1 mg/mL MTT solution prepared in phosphate-buffered saline (PBS). The formazan crystals were solubilized with isopropanol, and the absorbance was spectrophotometrically measured at the 595 nm wavelength using Thermo Appliskan microplate reader. The cell culture medium was taken after 24 h and 72 h and was used to measure the nitric oxide (NO) level by Griess test and for lactate dehydrogenase (LDH) release assay. For the Griess assay, cell culture supernatants were mixed with equal volumes of Griess reagent represented by a stoichiometric solution (*v*/*v*) of 0.1% naphthyl ethylenediamine dihydrochloride and 1% sulphanilamide in 5% H_3_PO_4_. The absorbance of the mix was read at 550 nm using the Thermo Appliskan microplate reader. Moreover, the amount of released LDH in the medium was measured with a cytotoxicity detection kit from Roche according to the manufacturer’s instructions, and the absorbance of the final mix was read at 450 nm.

### 2.6. Antimicrobial Performance

The obtained coatings were studied *in vitro* for their antimicrobial and anti-biofilm performance through the quantitative estimation of colony forming units (CFU/mL), a method allowing the assessment of viable cells embedded in the *S. aureus* bacterial biofilm (ATCC, Manassas, VA, USA) developed on the manufactured surfaces. The samples were priorly sterilized by UV exposure for 20 min on each side. Coated and uncoated specimens were placed in 6-well plates containing 2 mL of Luria Broth (LB) medium and inoculated with 50 µL bacterial suspensions of 0.5 McFarland standard density (1.5 × 10^8^ CFU/mL). The plates were left to incubate for 24 h then were washed with PBS solution; the culture medium was discarded and replaced with fresh LB medium. After consecutive incubation times (i.e., 24, 48, and 72 h), all specimens were rinsed with PBS and introduced individually into sterile tubes containing 1 mL of PBS. The biofilm-forming bacterial cells were dispersed by vigorously vortexing each tube for 30 s. Serial ten-fold dilutions were prepared from each obtained solution. Then, 10 µL from each dilution were placed on Petri plates containing LB agar, and after 24 h of incubation, the viable cells were counted at the optimal dilution (>30 and <300 colonies per plate) [49,50]. All the experiments were performed in triplicate [49].

### 2.7. Statistical Analysis

The *in vitro* assays were performed in triplicates, and the results were presented as the mean ± standard deviation (SD) of three independent experiments. The statistical significance was analyzed by one-way analysis of variance (ANOVA) followed by a Bonferroni post-hoc test for multiple comparisons using GraphPad Prism 9 software. A value of *p* less than 0.05 was considered significant.

## 3. Results

### 3.1. Physicochemical Characterization of HA and MgP Powders

**Figure 1:**
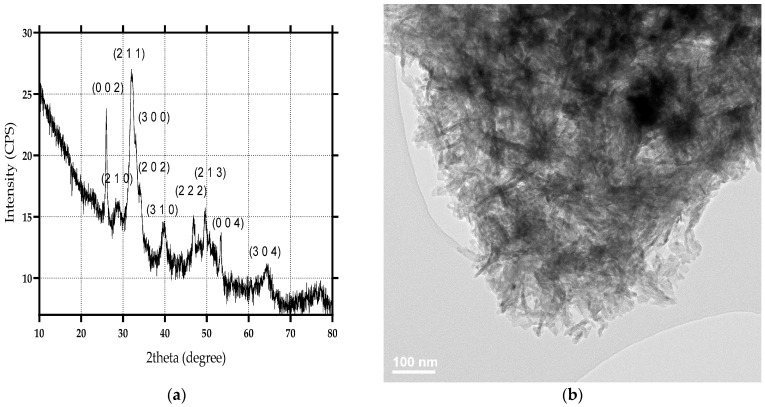

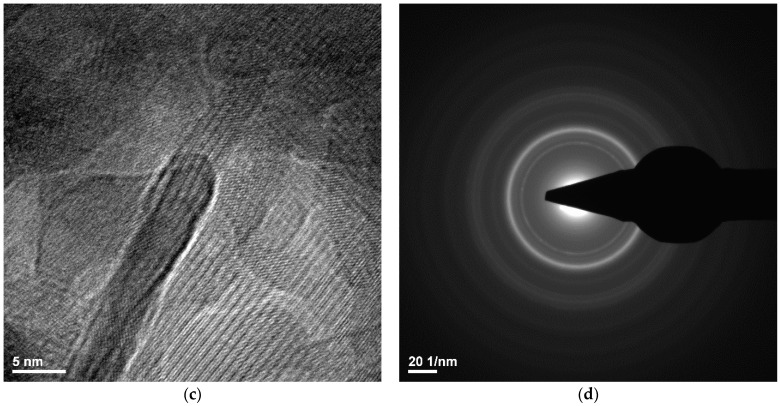
X-ray diffractogram (**a**), TEM image (**b**), HR-TEM image (**c**), and SAED pattern (**d**) for HA nanopowder.

The HA nanoparticles were evaluated through XRD, SEM, TEM, SAED, and IR. According to the obtained X-ray diffractogram, the main diffraction peaks correspond to the Miller indices specific for HA in the hexagonal crystal system (JCPDS no. 00-064-0738 [50,51,52]) (Figure 1a). Thus, the formation of HA as the unique crystalline phase was confirmed. Furthermore, the TEM images confirm the HA nanoparticles’ agglomeration tendency and their rod-like morphology, with lengths varying from 20–40 nm and widths up to 5 nm (Figure 1b,c). The crystallinity of the obtained HA nanoparticles was further proven through the non-diffused rings within the SAED patterns. The SAED Miller indices correspond to the ones identified within the diffractograms, thus demonstrating the purity of the synthesized HA.

Similarly, the MgP nanopowders were characterized through XRD, TEM, and SAED. Results were published elsewhere [53]. The acquired diffractogram confirms the formation of magnesium phosphate in the P21/n monoclinic system through the specific diffraction peaks and the corresponding Miller indices (JCPDS no. 00-033-0876 [54,55]). Moreover, the agglomeration tendency and the polyhedral shape of the MgP particles can be observed through TEM images. The SAED patterns reveal the presence of spots, which are generally characteristic of monocrystalline materials [53].

**Figure 2:**
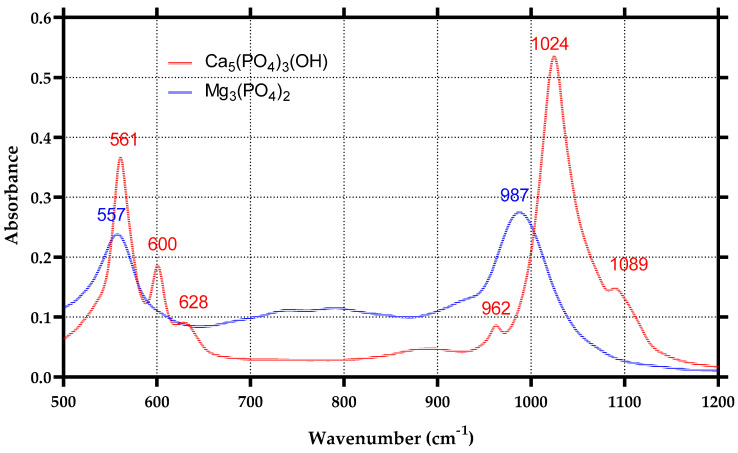
IR spectra of HA and MgP.

The IR patterns of MgP show the band at 987 cm^−1^, which is due to the P-O stretching vibrations, whereas the band at 557 cm^−1^ is due to the O-P-O bending vibrations (Figure 2).

The HA has distinct absorption bands (Figure 2). The bands at 600 and 561 cm^−1^ are components of the PO_4_ ν4 vibrational mode. The 628 cm^−1^ vibrational band is assigned to the liberational mode of the hydroxyl group. The PO_4_ ν1 vibrational band is shown at 962 cm^−1^, and the PO_4_ ν3 vibrational bands are at 1089 and 1024 cm^−1^ [56,57,58].

### 3.2. Physicochemical Characterization of Composite Coatings

In order to examine the compositional integrity of laser-processed materials, we conducted comparative IR studies on the drop-cast (DC) samples vs. MAPLE coatings.

**Figure 3:**
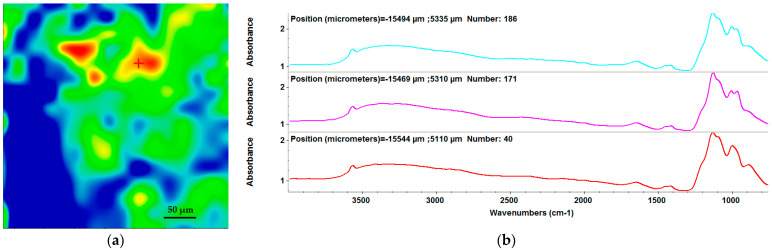
IR map (**a**) and corresponding FT-IR spectra (**b**) assigned to the phosphate group for the drop-cast reference material.

The IR map for the drop-cast sample was acquired by monitoring the stretching vibration of the P-O bond within the phosphate group (at ~1100 cm^−1^) associated with the presence of HA and MgP (Figure 3) [59,60].

**Figure 4:**
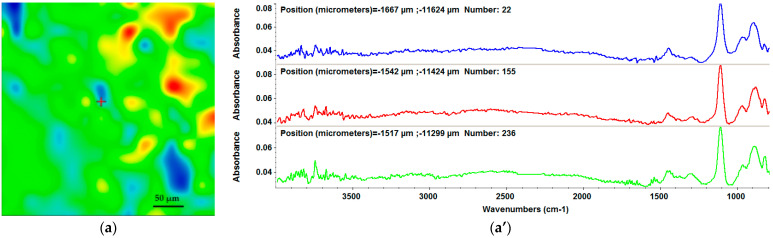

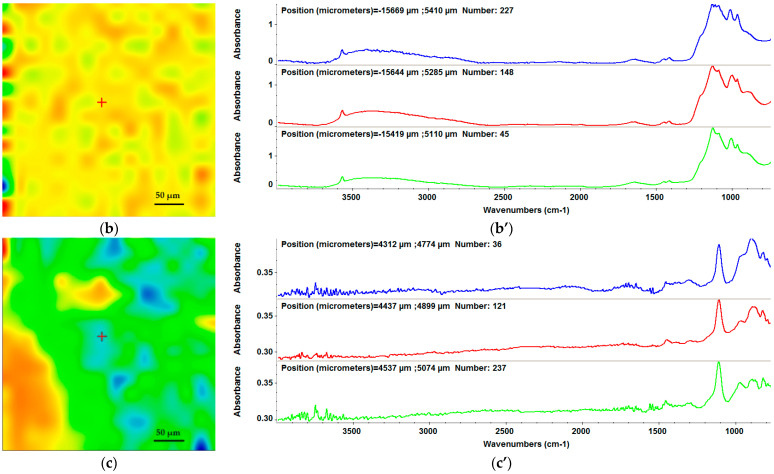
IR maps (**a**–**c**) and corresponding FT-IR spectra (**a’**–**c’**) for HA/MgP/BMP4 coatings obtained at (**a**) 300 mJ/cm^2^, (**b**) 400 mJ/cm^2^, and (**c**) 500 mJ/cm^2^.

From the IR spectra of samples subjected to drop-cast (Figure 3b) and MAPLE deposition at 400 mJ/cm^2^ (Figure 4b), we can observe a stoichiometric transfer.

There are multiple color zones that demonstrate the variation of the amount of the HA/MgP/BMP4 coating (red is associated with high absorbance intensities and, consequently, high amounts of the nanocoating, whereas blue is characteristic of low absorbance intensities).

Thus, the HA/MgP/BMP4 coatings obtained through the MAPLE technique at 300, 400, and 500 mJ/cm^2^ laser fluences were also examined by IR mapping and FT-IR (Figure 4). The FT-IR spectra present an overlapping sum of absorption bands earlier discussed (Figure 2). IR maps were created based on the intensity of the PO_4_ absorption band (highest peak).

From the IR maps, one can observe structural modifications for the depositions performed at 300 and 500 mJ/cm^2^, where the quantity of the material is increasing from blue to red areas (Figure 4a,c), which is in accordance with the FT-IR spectra (Figure 4a’,c’). Moreover, the FT-IR spectra showed the integrity of the phosphate group at the 400 mJ/cm^2^ laser fluence (Figure 4b’), being similar to the one pertaining to the dropcast. The laser fluence of 400 mJ/cm^2^ was therefore considered optimal, and the as-fabricated coatings were subjected to further investigations

Considering these results in association with the classical IR analysis, it can be stated that the MAPLE deposition technique did neither damage the functional groups nor induce changes to the chemical structure of the raw material. After this stage, CXF was nebulized on the coatings surface to prepare a multifunctional surface and provide an antimicrobial effect.

**Figure 5:**
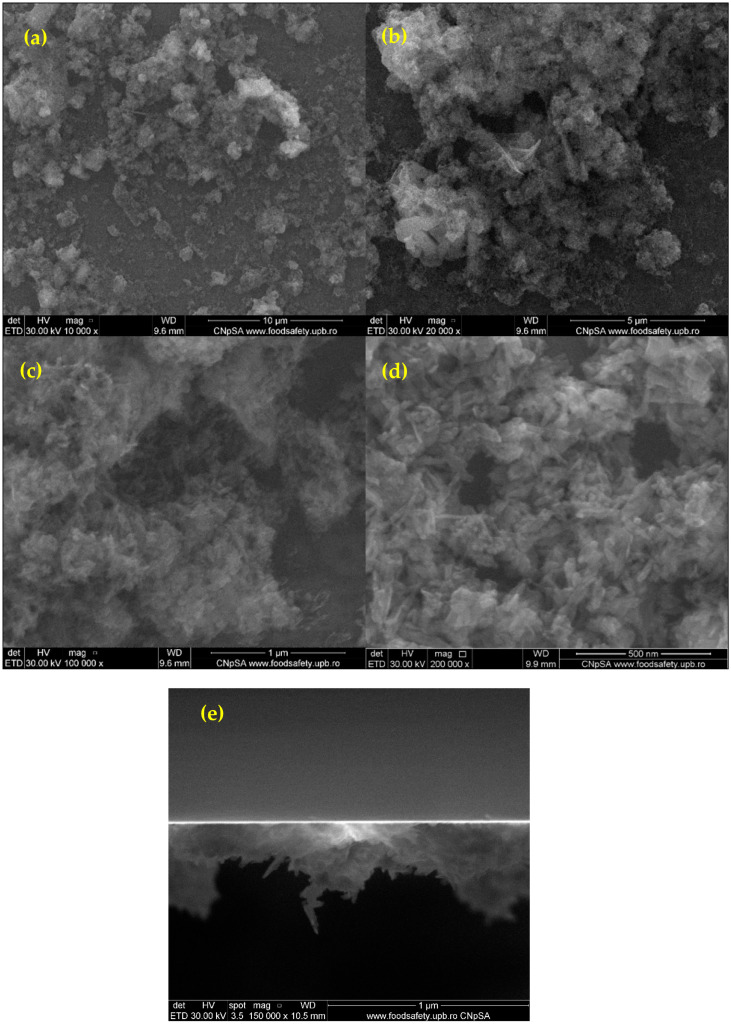
SEM images at various magnifications (**a**—10,000×, **b**—20,000×, **c**—100,000×, **d**—200,000×) and cross-section (**e**) for the HA/MgP/BMP4 coatings.

Furthermore, the HA/MgP/BMP4 coatings were subjected to SEM analysis to investigate the surface morphology of the prepared coating (Figure 5). In this context, the SEM images reveal a relative distribution of the coating on the substrate with the presence of both HA and MgP powders. The coating has several agglomerates, and the thickness of the tested coating varies from 200 to 500 nm (Figure 5e).

### 3.3. Cytocompatibility Evaluation on Osteoblasts

In this study, the bioactive coatings were further studied for their cytocompatibility and antimicrobial properties. The coatings’ bioactivity was achieved by adding different types of bioactive molecules, i.e., growth factors that could considerably enhance the formation of the natural bone, a key stage in the tissue-healing process, and one antibiotic that could prevent microbial colonization and the occurrence of periprosthetic infections. The growth factor molecule used in our study is represented by BMP4 (SigmAldrich, Saint Louis, MO, US), which is naturally located within the interstitial tissue of the bone, stimulating bone regeneration [61,62,63]. BMPs have been either immobilized at the surface of the orthopedic implant or loaded in implant coatings, this last approach exhibiting the advantages of an increased incorporation rate and bioactivity, as well as a slow, localized release. BMP4 activity could be assessed *in vitro* by determining the morphology, attachment, proliferation, and differentiation of different types of cells on bioactive implant surfaces. In our study, we tested the interaction between the HA/MgP/BMP4 coatings and the murine osteoblasts belonging to the MC3TE-E1 cell line.

**Figure 6:**
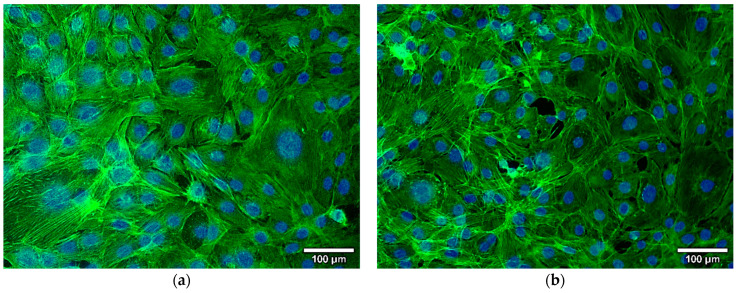
Fluorescence images of actin filaments of MC3T3-E1 osteoblasts cultured at 24 h on the uncoated control (**a**) and on the composite HA/MgP/BMP4 coatings (**b**).

Figure 6 depicts the fluorescence microscopy images of the cells grown on the HA/MgP/BMP4 coatings labeled with phalloidin-FITC. It can be stated that the HA/MgP/BMP4 coatings did not induce any morphological changes in MC3TE-E1 osteoblasts, which exhibited normal shapes suggesting a healthy status. The osteoblast-specific architecture and high monolayer confluency could be observed on the surface of coatings, with central nuclei and elongated actin filaments.

**Figure 7:**
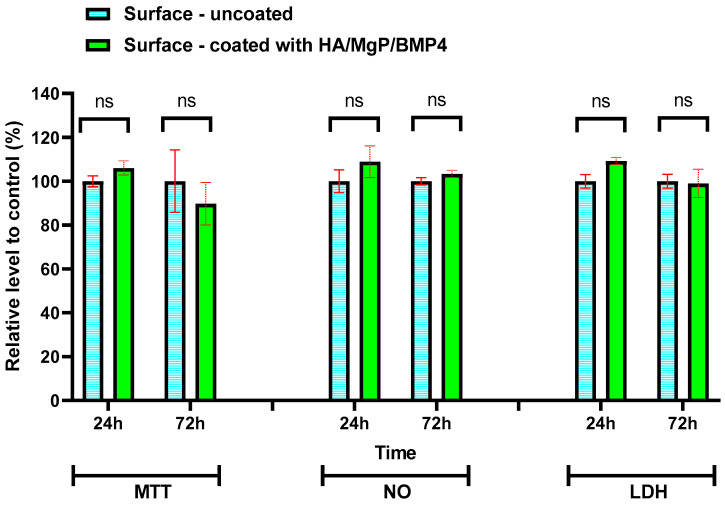
Cellular viability, NO level, and LDH release after 24 h and 72 h of MC3T3-E1 osteoblasts’ growth on the control surface and in contact with HA/MgP/BMP4 coating; ns = not significant, *p* > 0.05.

Moreover, the MTT assay was performed for the HA/MgP/BMP4 coatings to evaluate the effect of BMP4 in terms of cellular viability (Figure 7). The percentage of live cells grown on the nanocoated surface was in the proximity of control values after 24 h and 72 h, demonstrating the release in the active form of the BMP4, which has been able to facilitate the cell attachment and maintain a normal osteoblast proliferation. In addition, the presence of MgP is very important, as it was shown that Mg is necessary for ATP synthesis and ATP-driven enzymatic reactions, favoring osteoblastogenesis [64].

The lack of cytotoxicity towards the MC3T3-E1 cells was also confirmed by the levels of NO and LDH release, which were comparable with those obtained for the control sample after both intervals of incubation, 24 h and 72 h. No significant inflammation and damage on the cell membrane (*p* > 0.05) were noticed during the 72 h incubation of cells on the tested coating. Thus, the *in vitro* results showed that this HA/MgP/BMP4 coating could be efficiently used for developing promising coatings for orthopedic implants that do not interfere with the osteointegration process. Taking into account that the coatings are very thin, the release of bioactive molecules for 72 h will have immediate positive effects on the cells coming into contact with the implant.

### 3.4. Antibiofilm Activity

One of the intended performances of the obtained bioactive coatings was to provide antimicrobial protection against the adherence and development of microbial biofilms. Due to their high tolerance to antibiotics and host immunity, once developed on the surface of a prosthetic device or implant, biofilms are very difficult, if not impossible, to be treated, leading in many cases to the removal of the affected implant [45]. Therefore, preventing biofilm formation on the implant surface remains a challenge for materials science and different medical specialties. One of the research directions is to develop novel materials with improved resistance to microbial colonization or bioactive implant coatings able to provide an antimicrobial effect in the vicinity of the implanted material by incorporating and then locally releasing conventional antibiotics or other antimicrobial agents.

Previous studies demonstrated the efficacy of antimicrobial coatings, such as those containing silver, gentamycin, and iodine, in the decrease of post-operative peri-prosthetic infection rates, thus having a positive impact on the prevention of periprosthetic infections [65] without any detectable adverse events or side effects [66].

In our study, we incorporated in our coatings the antibiotic CXF and investigated its ability to inhibit the biofilm’s early stages but also *S. aureus* development and maturation.

*S. aureus* is one of the major threats regarding antimicrobial resistance, being included in the ESKAPE(E) pathogens list [*Enterococcus faecium*, *Staphylococcus aureus*, *Klebsiella pneumoniae*, *Acinetobacter baumannii*, *Pseudomonas aeruginosa*, *Enterobacter* sp. (*Escherichia coli*)] and also listed by the WHO as one of the bacterial species requiring high priority for the development of novel antibiotics. Besides this genetic resistance, *S. aureus*, *S. epidermidis*, and *P. aeruginosa* are the most common biofilm-forming bacteria, being found in up to 75% of the medical devices associated with biofilms [67]. *S. aureus* is involved in 12–23% of periprosthetic infections [67]. Moreover, *S. aureus* is able to enter, survive, and proliferate in non-phagocytic cells, such as epithelial and osteoblast cell types, being a potential source of invasive infections [68].

To confirm the antimicrobial effect of our coating, we used a culture-based approach to quantify the viable cells embedded in the biofilm formed on the surface of coated materials at different times (after 24 h, to evidence the ability of the antibiotic to reduce surface colonization and thus biofilm initiation, after 48 h, to reveal the capacity of the coating to delay the biofilm development and after 72 h, to highlight the potential inhibitory effect of the coating against mature biofilms).

**Figure 8:**
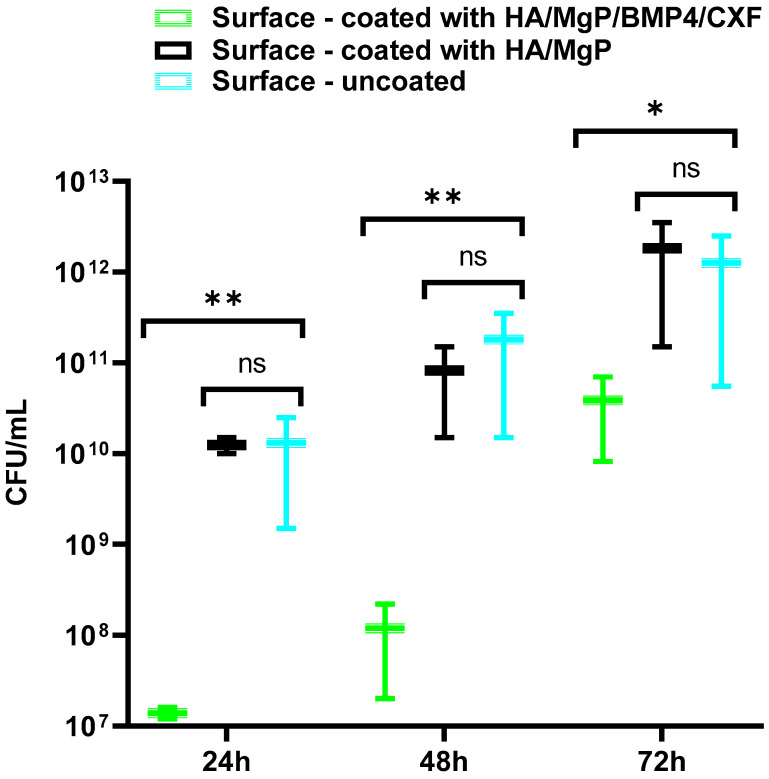
Antibiofilm efficiency assessment of the uncoated and coated surfaces against *S. aureus* at 24 h, 48 h, and 72 h; *—*p* ≤ 0.05; **—*p* ≤ 0.005; ns = not significant, *p* > 0.05.

The results presented in Figure 8 show that the obtained nanocoating is releasing the antibiotic in an active form, and it protects the surface colonization by killing the planktonic cells attached to the implant surface. The dynamics of the viable cell counts suggests a rapid burst of high concentrations of antibiotics within the first 48 h, as demonstrated by the very significant decrease (by ~3 logs) of the number of viable cells recovered from the coated surface after 24 h in comparison with the uncoated and coated surfaces with the biodegradable cement without antibiotic. The significant decrease in the number of viable cells harvested from the colonized samples after 48 h demonstrates that the antibiotic is still being released from the coating and delays the biofilm development. After 48 h, the protective effect of the antibiotic is less evident but persists even after 72 h. This could be explained by the lower inhibitory effect of the released antibiotic combined with the biofilm maturation, the phenotype of biofilm embedded bacterial cells being much more tolerant to antibiotics.

The antibiofilm efficiency of HA/MgP/BMP4/CXF is much higher than that of the surface coated only with HA-BMP and the uncoated surface. This is a very significant result because the loading of the antibiotic in the coating prevents the numerous side effects of the systemic antibiotic administration, such as the need for high doses, low bioavailability at the implant site, or induction of gut dysbiosis that could affect the entire body homeostasis [69].

Moreover, there were recently reported point-of-care coating technologies that could be performed in the operating room in less than 10 minutes and can incorporate any desired antibiotic, thus providing personalized approaches depending on the patient’s condition and the local epidemiological context regarding bacterial resistance. The chosen antibiotic or mix of antibiotics regularly must ensure a broad spectrum and provide a local concentration equal to the minimal inhibitory concentration [69]. Point-of-care coating technologies have already been tested to generate amphiphilic polymeric coatings, displaying *in vivo* efficacy with or without systemic antibiotics [70].

## 4. Conclusions

In the present study, we combined the advantages of two ceramic materials with well-known benefits within bone tissue applications, namely HA and MgP, with those of the BMP4 growth factor and of a large spectrum antibiotic for developing bioactive coatings for bone implants. The experimental study demonstrated the successful synthesis of HA and MgP powders, followed by optimal material deposition by the MAPLE technique at 400 mJ/cm^2^ laser fluence. Furthermore, the composite coating proved to exhibit a significant antimicrobial and antibiofilm effect, especially in the first 48 h of colonization, and biocompatibility in relation to osteoblast cells, as revealed by their normal morphology, viability, membrane integrity, and actin cytoskeleton, thus confirming the potential of the present system for developing novel implant coatings.

## Data Availability

Not applicable.
